# Value of Post-transfer Day-12 Beta Human Chorionic Gonadotropin Levels for Pregnancy Outcome Prediction of Intracytoplasmic Sperm Injection Cycles

**DOI:** 10.4274/balkanmedj.2016.1769

**Published:** 2017-09-29

**Authors:** İnci Kahyaoğlu, Berfu Demir, Sezin Ertürk Aksakal, İskender Kaplanoğlu, Leyla Mollamahmutoğlu

**Affiliations:** 1 Department of Obstetrics and Gynecology, University of Health Sciences, Ankara Etlik Zübeyde Hanım Women’s Health Training and Research Hospital, Ankara, Turkey; 2 Center of Assisted Reproduction, University of Health Sciences, Ankara Etlik Zübeyde Hanım Women’s Health Training and Research Hospital, Ankara, Turkey

**Keywords:** Beta human chorionic gonadotropin, intracytoplasmic sperm injection, clinical pregnancy, ongoing pregnancy

## Abstract

**Background:**

Several markers were studied previously in order to predict the pregnancy outcome of assisted reproductive techniques; however, serum beta human chorionic gonadotropin was found title be the most predictive marker.

**Aims:**

To evaluate the value of serum beta human chorionic gonadotropin levels in discriminating biochemical and clinical pregnancies 12 days after embryo transfer, while determining the factors predicting ongoing pregnancy was established as the secondary aim.

**Study Design:**

Retrospective cross-sectional study.

**Methods:**

A total of 445 pregnant cycles were retrospectively analysed in 2359 embryo transfer cycles. Patients were divided into two groups according to the outcome of pregnanctitley: biochemical and clinical.

**Results:**

The cut-off value of beta human chorionic gonadotropin levels on day 12 in predicting clinical pregnancies was 86.8 IU/mL with 65.1% sensitivity and 74.7% specificity [CI: 0.76 (0.71-0.81). Receiver operating characteristic curve analysis revealed different cut-off values for embryo transfer days (57 mIU/mL for day 3 embryo transfer CI: 0.59-0.79 and 87 mIU/mL for day 5 embryo transfer, CI: 0.74-0.86). Subgroup analysis of clinical pregnancies revealed a significant difference between ongoing pregnancies and early fetal losses regarding duration of infertility (81.3±54.4 vs. 100.2±62.2 months), serum oestradiol on hCG day (2667.4±1276.4 vs. 2094.6±1260.5 pg/mL), number of transferred embryos (1.9±0.8 vs. 1.5±0.7) and the prevalence of diminished ovarian reserve as an indication (2.3% vs 12.2%).

**Conclusion:**

Beta human chorionic gonadotropin levels on day 12 following embryo transfer provide an important parameter for the prediction of clinical pregnancy; however, other stimulation parameters are indicated in the prediction of ongoing pregnancies.

Infertility has been referred to as one of the biggest sources of stress in a patient’s life (1) and is regarded as a psychological trauma for most couples (2). The highest level of stress experienced by the patient is demonstrated to be at the stage of the pregnancy test (3). Moreover, the time interval after the test and the confirmation of a viable intrauterine pregnancy by ultrasonography is considered to be extremely or very stressful by 46% of patients (4). When taken together with the increased risk of early pregnancy loss in pregnancies achieved by assisted reproduction compared to spontaneous pregnancies (5), this time interval can be considered an emotionally challenging period for couples. Therefore, the early prediction of pregnancy outcome is of great importance for patients in order to prepare them for an adverse pregnancy outcome and also for the clinician to counsel and manage the patient accordingly.

Several markers were studied previously in order to predict the pregnancy outcome of in vitro fertilisation (IVF) pregnancies, such as serum oestradiol, progesterone (6), CA-125 (7) or inhibin (8). However, beta human chorionic gonadotropin (β-hCG) was found to be the most predictive marker (9,10,11,12,13). A direct correlation has been shown between the level of β-hCG after implantation and pregnancy outcome (9,10,12,13,14). Low levels of serum β-hCG in early pregnancy predict poor pregnancy outcome (9,15,16,17,18,19,20,21,22).

The primary aim of this study was to evaluate the value of serum β-hCG levels in discriminating biochemical and clinical pregnancies 12 days after embryo transfer (ET) and to determine the factors predicting whether ongoing pregnancy was established as the secondary aim. Improvement of patient counselling by giving reliable predictive information and alleviation of the anxiety of the patient with these results was intended.

## MATERIALS AND METHODS

A computer based database of 2359 IVF cycles performed between March 2007 and May 2014 at an IVF clinic of a tertiary teaching and research hospital were reviewed retrospectively. ET cycles with available data on levels of serum β-hCG 12 days after ET were included in the study. Exclusion criteria were determined as follows: frozen-thawed embryo cycles, cycles with cancelled ET because of failed fertilisation, failed cleavage and cycles lost to follow-up. Diminished ovarian reserve was defined as maternal age at or over 40 years, previous poor ovarian response with three or less oocytes obtained with conventional stimulation, antral follicle count (AFC) below 7 follicles or AMH below 1.1 ng/mL. Male factor infertility was determined according to semen analysis; azoospermia, severe oligozoospermia (<5x106/mL) and retrograde ejaculation was regarded as an indication for assisted reproductive techniques (ART). At the beginning of the study, approval was obtained from the institutional review board (University of Health Sciences, Ankara Etlik Zübeyde Hanım Women’s Health Training and Research Hospital number: 27.02.2014/171). Informed consent was taken from all patients. Serum β-hCG levels 12 days after ET, demographic characteristics [age, body mass index, basal serum follicle stimulating hormone (FSH), total AFC, duration of infertility, reason for infertility], controlled ovarian stimulation parameters and embryology data were compared between biochemical and clinical pregnancies. Factors affecting ongoing pregnancies and early fetal loss were also analysed. 

Luteal long gonadotropin-releasing hormone agonist, microdose flare-up or antagonist protocols were used for controlled ovarian stimulation. Either pure recombinant FSH or human menopausal gonadotropin were used and individualised gonadotropin doses were used for each patient. Serial transvaginal ultrasound evaluation and serum oestradiol levels were used to monitor the cycles. When at least three follicles showed a mean diameter of 17 mm, recombinant hCG 250 μg (Ovitrelle, Serono, İstanbul, Turkey) was administered and oocyte pick up procedures were performed 35.5-36 hrs after the hCG injection by transvaginal ultrasound-guided aspiration. Intracytoplasmic sperm injection (ICSI) procedure was done for all the patients. ET was performed on the second, third or fifth day following ICSI. The number of embryos transferred was determined according to the patient’s age, the number of previous attempts and embryo quality. Luteal phase support was given by vaginal progesterone (Crinone 8% gel, Serono, UK) twice daily. From October 2013, intramuscular progesterone 100 mg daily (Progestan 50 mg ampul, Koçak, İstanbul) was combined with vaginal progesterone until the serum β-hCG test. Luteal phase support was given until 12 weeks of gestational age.

Blood was drawn 12 days after the ET to determine the serum quantitative β-hCG concentrations. Pregnancy was defined if the hCG serum concentration was higher than 10 IU/L. If the day 12 β-hCG test was positive, a second serum sample was taken 48 h later for β-hCG concentration, in order to discriminate between a viable intrauterine pregnancy and a possible ectopic pregnancy. An ultrasound was then performed 4 weeks after the ET to verify the number of gestational sacs and cardiac activity. Serum analysis was performed with analysers utilising the Beckman Coulter Access total β-hCG calibrated to the World Health Organisation Third International Standard 75/537. Biochemical pregnancy was defined as pregnancy detected by hCG measurement without any gestational sac seen on ultrasound and clinical pregnancy was diagnosed by the detection of fetal heart rate. Ongoing pregnancy was defined as one that proceeded beyond 12 weeks gestation.

### Statistical analysis

IBM SPSS Statistics Software (21.0, SPSS Inc., Chicago, IL, USA) was used for statistical analysis. The distribution of variables was tested by Shapiro-Wilks test. Student’s t-test was used for variables with normal distribution whereas the Mann-Whitney U test was used for data with skewed distribution. Chi-square test was used for the comparison of proportions. Continuous variables were presented as mean ± standard deviation. A receiver operating characteristic (ROC) curve was used to compare sensitivity and specificity at each β-hCG value to predict clinical and ongoing pregnancies. Sensitivity, specificity, area under the ROC curve and 95% CIs were calculated for each of the estimates. Logistic regression was used to predict the chance of having an ongoing pregnancy after ET. Associations of initial β-hCG, age, total AFC, maximum E2 level and endometrial thickness at ET with pregnancy outcome were evaluated with logistic regression models. Power calculation was obtained using MedCalc 11.1.1.0 (MedCalc® Statistical Software, Belgium). In a prior study, Urbancsek et al. (14) reported that a β-hCG difference of 66 IU was significant in day 11. Using this rate, assuming a margin of equivalence of 20% with significance set at 0.05, our study would be adequately powered (at least 80%) with 88 patients. Statistical significance was defined as p<0.05.

## RESULTS

**FIG. 1.**
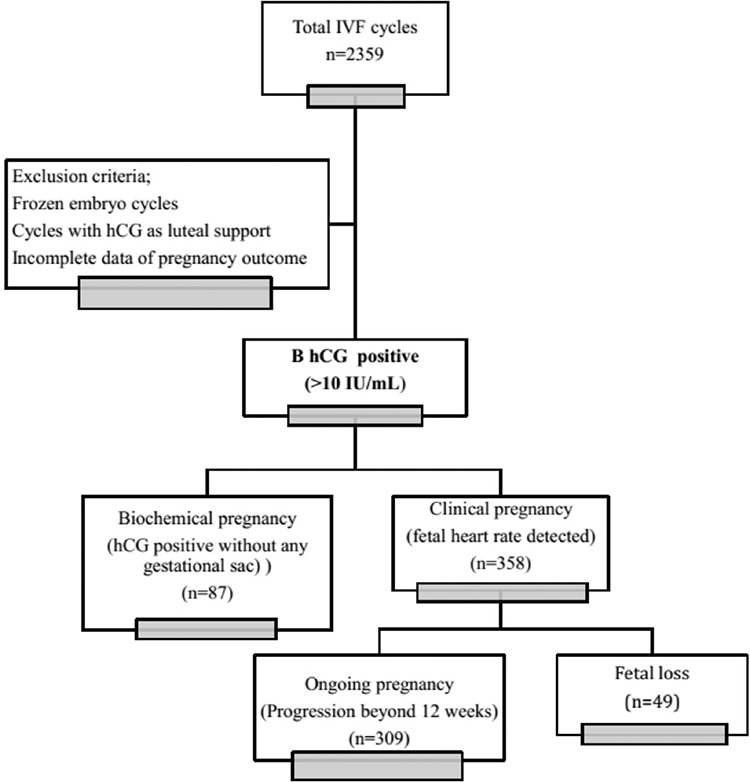
Flow chart of the study.

A total of 445 pregnancies fulfilled the inclusion criteria. Of the 445 pregnancies, 87 (19.6%) were biochemical and 358 (80.4%) were clinical pregnancies. Out of 358 clinical pregnancies, 86.3% (309 of 358) progressed beyond 12 weeks of gestation (ongoing) while the remaining 13.7% (49 of 358) resulted in early fetal loss (Figure 1).

Day 12 hCG levels differed significantly between biochemical and clinical pregnancies (p<0.001). No significant difference was found between biochemical and clinical pregnancies regarding demographic characteristics (p>0.05). With respect to stimulation parameters, E2 on hCG day, number of retrieved oocytes, number of mature oocytes and E2 on ET day were significantly higher in clinical pregnancies when compared to biochemical pregnancies (p<0.05). The day and number of transferred embryos were comparable between the two groups (Table 1).

**FIG. 2.**
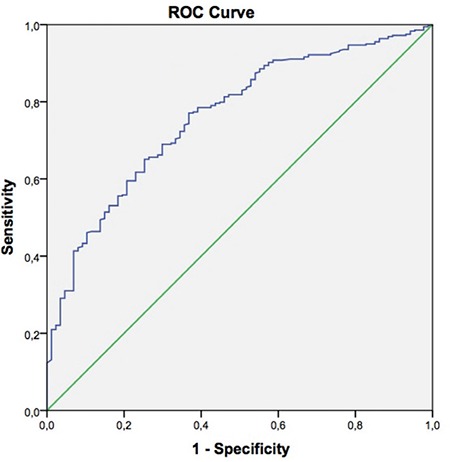
Receiver-operator curve analysis for the performance of serum beta human chorionic gonadotropin 12 days after embryo transfer in the prediction of clinical pregnancies.

**FIG. 3.**
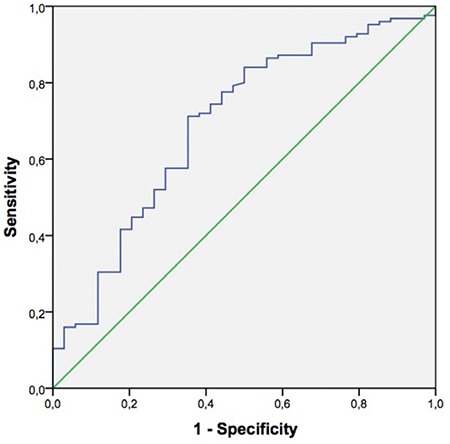
Receiver-operator curve analysis for the performance of serum beta human chorionic gonadotropin 12 days after embryo transfer in the prediction of clinical pregnancies for day 3 transfers.

**FIG. 4.**
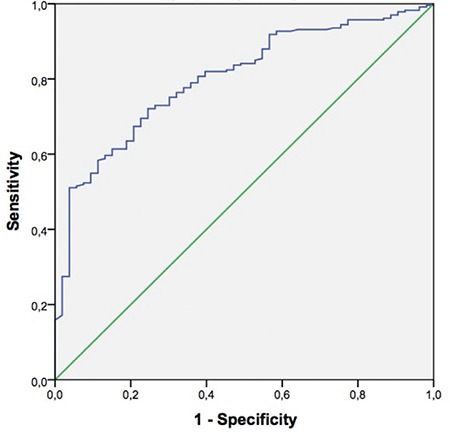
Receiver-operator curve analysis for the performance of serum beta human chorionic gonadotropin 12 days after embryo transfer in the prediction of clinical pregnancies for day 5 transfers.

ROC curve analysis revealed a β-hCG threshold value of 86.8 IU/mL with 65.1% sensitivity and 74.7% specificity for prediction of clinical pregnancy [AUC (CI): 0.76 (0.71-0.81)] (Figure 2). When serum β-hCG levels 12 days after transfer were evaluated according to the transfer day, the values that were most reliable in predicting clinical pregnancy were 57 mIU/mL for day 3 transfer cycles (sensitivity 71.2%, specificity 64.7%, CI: 0.59-0.79) (Figure 3), and 87 mIU/mL for day 5 transfers (sensitivity 71.2%, specificity 75.5%, CI: 0.74-0.86) (Figure 4).

When demographic and ovarian stimulation characteristics of the patients in subgroups of clinical pregnancies (ongoing pregnancies and early fetal loss) were compared, no significant difference was found with respect to serum β-hCG levels on day 12 of ET (p>0.05) (Table 2). No significant difference was found between two groups regarding age, baseline FSH, or total AFC (p>0.05). The duration of infertility was significantly shorter in ongoing pregnancy group when compared to early fetal loss group (p=0.046). Regarding the indications of ART treatment, the rate of diminished ovarian reserve was significantly higher in the early fetal loss group (12.2%) than in the ongoing pregnancy group (2.3%) (p=0.002). With respect to stimulation parameters, E2 on hCG day and number of transferred embryos were significantly higher in the ongoing pregnancy group than in early fetal loss group (p=0.02 and p=0.001, respectively).

According to the results of logistic regression analysis, β-hCG and E2 levels on hCG day were found to be significant variables correlated with ongoing pregnancy [OR: 1.0042 (95% CI: 1.0025; 1.0058) and OR: 1.00035 (95% CI: 1.00016; 1.00053) respectively, (p<0.001)]. A 1-unit increase in β-hCG level is associated with an increase in the odds of success by a factor of 0.042 while E2 is associated with an increase of 0.0035 (Table 3).

## DISCUSSION

The results of the present study demonstrated that serum β-hCG levels on day 12 of ET have a predictive value in discrimination between biochemical and clinical pregnancies. This value was not found to be a predictor of successful pregnancy continuation beyond 12 weeks of gestation. Day 12 β-hCG levels were demonstrated to vary according to the stage of embryonic development at the time of transfer. Higher β-hCG threshold levels for clinical pregnancies based on ROC curve analysis were found for day 5 transfers compared to day 3 transfers.

Serum β-hCG levels represent trophoblastic mass and function, which play a critical role in early pregnancy, as they sustain the corpus luteum and have a role in endometrial regulation, placental syncytium formation and implantation (22). It has been proposed that the absolute β-hCG value may reflect the quality of implantation (23) and may be used as a marker of implantation success (24). Although various serum markers were investigated as predictors of pregnancy outcome after assisted reproduction, serum β-hCG was shown to be a reliable and the earliest indicator of pregnancy outcomes in IVF cycles (25,^26^). Different cut-off values ranging from 50 IU/L to 76 IU/L for 11 to 14 days after ET were reported in the literature to predict viable and non-viable pregnancies with varying sensitivity and specificities (19,20,21,27). Similar to these findings, our results also revealed a slightly higher day 12 β-hCG cut-off value of 86.8 IU/mL discriminating best between biochemical and clinical pregnancy with a sensitivity of 65.1% and specificity of 74.7%. On the other hand, there are conflicting results in the literature regarding cut-off levels with respect to the day of transferred embryos. Kumbak et al. (10) evaluated serum β-hCG values 12 days after transfer of day 3 and day 5 embryos and showed that the values that were most reliable in predicting ongoing pregnancy to be 98 mIU/mL for day 3 transfer cycles, and 257 mIU/mL for day 5 transfers. They concluded that at a fixed time after transfer, the blastocyst can produce higher amounts of β-hCG than cleavage stage embryos, depending on the higher number of viable trophoectoderm cells that they have at that time point. Two studies are in line with this result, reporting higher β-hCG values for day 5 embryos than day 3 embryos (26,28). Kathiresen et al. (26) reported cut-off values of 78 IU/L and 160 IU/L for day 3 and day 5 embryos, respectively, 15 days after transfer for prediction of ongoing pregnancies. It was stated in that report that the difference between initial β-hCG levels may result from different developmental stages of embryos transferred, which may have different developmental potential and eventually may impact implantation rates. Papageorgiou et al. (28) demonstrated slightly lower cut-off levels, 32 IU/L and 173 IU/L, for day 3 and day 5 embryos, respectively, when blood was drawn 16 days after fertilisation, supporting the hypothesis that blastocysts produce higher levels of β-hCG due to their more advanced stage of development. In contrast, Zhang et al. (29) demonstrated lower initial serum β-hCG after day 5 transfers when compared to day 3 transfers (75±54 vs. 62±41), suggesting that embryo development and implantation may be impaired by the additional two days of culture. In line with the previous studies, the results of our study also demonstrated higher day 12 cut-off values of β-hCG for day 5 embryos than day 3 embryos for the prediction of clinical pregnancy (86.9 IU/mL vs. 57.6 IU/mL, respectively).

Poor ovarian responders revealed poor pregnancy outcome even if the initial β-hCG results were positive. SART data demonstrated lower pregnancy rates in women with diminished ovarian reserve among other indications of IVF, irrespective of age. The pregnancy rate in women with diminished ovarian reserve is 34.1% below 35 years old, however the rates are 50.5% and 49.2% in male infertility and ovulatory dysfunction, respectively (30). The results of the present study are also in line with these data, revealing clinical pregnancy rates of 82.2%, 81% and 68.4% in patients with the indications of unexplained infertility, male infertility and diminished ovarian reserve. Significantly higher levels of E2 on hCG day both in clinical and ongoing pregnancies also support the higher chance of pregnancy in patients with good response to ovarian stimulation. Although the higher number of transferred embryos increases the chance of ongoing pregnancy, it could be hypothesised that implantation potentials of better quality embryos from good responders may also make a contribution to these results. Moreover, pregnancy loss in patients with diminished ovarian reserve was also noted to be significantly higher for all age groups when compared to patients with normal ovarian reserve. The vast majority of losses were reported to be in early gestational weeks, i.e., prior to the detection of a clinical pregnancy. On the other hand, as oocytes of the poor responders are the last oocytes of the ovarian pool and at increased risk of chromosomal abnormalities, they are suggested to be poor quality. Fetal aneuploidy resulting from these oocytes can result in early spontaneous abortions. The rates of clinical and ongoing pregnancy rates among total pregnant women with male infertility were 80.9% and 68.1% while it was 82% and 74% for unexplained infertile women in our study. However, there was a steep decline in women with decreased ovarian reserve from 68.4% to 36.8%, which confirms the increased first trimester loss rate in this group of patients.

The main limitation of this study was its retrospective nature, although a large number of cases were included in the study.

In conclusion, β-hCG levels 12 days after ET have value for the prediction of clinical pregnancy, but not for ongoing pregnancy. The cut-off values vary depending on the day of ET, with higher values seen with blastocyst transfers. However, the probability of progression beyond 12 weeks of gestation is lower in poor responders than any other indications of ART, once clinical pregnancy is detected.

## Figures and Tables

**Table 1:**
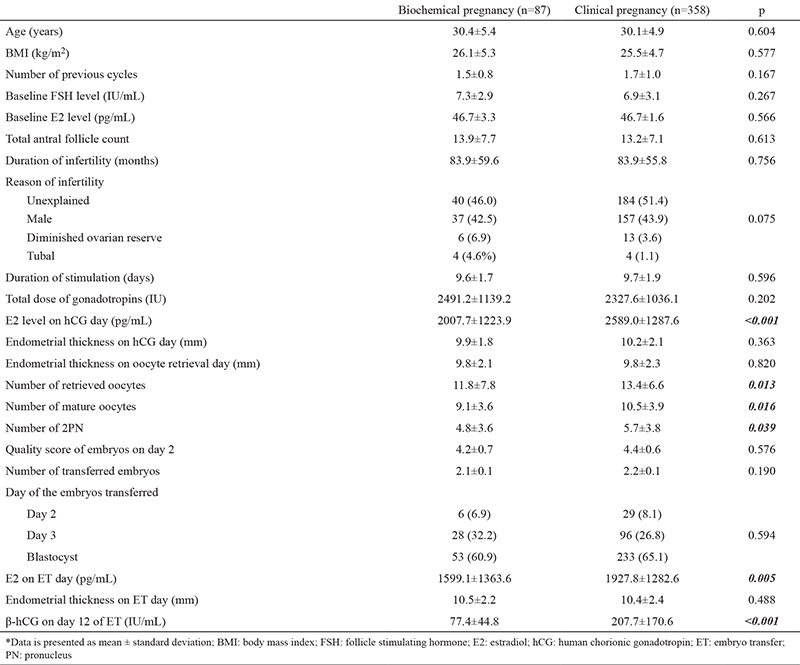
Demographic and controlled ovarian stimulation parameters of biochemical and clinical pregnancies

**Table 2:**
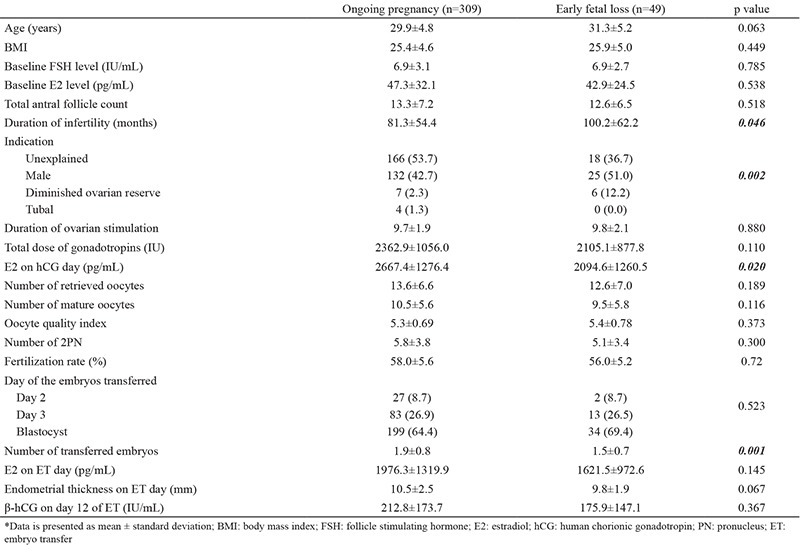
Demographic characteristics and results of controlled ovarian stimulation parameters in the ongoing pregnancy and early fetal loss groups

**Table 3:**
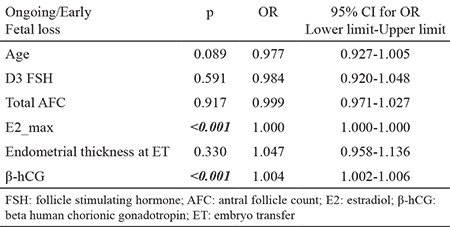
Summary of logistic regression analysis of variables predicting ongoing pregnancies
